# Non-communicable disease risk factor profile among public employees in a regional city in northern Ethiopia

**DOI:** 10.1038/s41598-018-27519-6

**Published:** 2018-06-18

**Authors:** Lemlem Weldegerima Gebremariam, Chifa Chiang, Hiroshi Yatsuya, Esayas Haregot Hilawe, Alemayehu Bayray Kahsay, Hagos Godefay, Loko Abraham, Yoshihisa Hirakawa, Hiroyasu Iso, Atsuko Aoyama

**Affiliations:** 1 Department of Public Health and Health Systems, Nagoya University School of Medicine, Nagoya, Japan; 2 Department of Public Health, Fujita Health University School of Medicine, Toyoake, Aichi Japan; 3 Tigray Health Research Institute, Mekelle, Ethiopia; 4 College of Health Sciences, Mekelle University, Mekelle, Ethiopia; 5 Tigray Regional Health Bureau, Mekelle, Ethiopia; 6 Ethiopian Pharmaceuticals Fund and Supply Agency, Addis Ababa, Ethiopia; 7 Public Health Graduate School of Medicine, Osaka University, Suita, Osaka Japan

## Abstract

The burden of non-communicable diseases (NCDs) is increasing in Ethiopia. This study aims to describe the prevalence of NCD risk factors of public employees in a regional city in northern Ethiopia. We conducted a cross-sectional epidemiological study targeting men and women aged 25–64 years employed by public offices in Mekelle. The prevalence was age-standardized to the Ethiopian 2007 population. Among the 1380 subjects (823 men and 557 women), 68.7% had less than 1 serving of fruits and vegetables per day, 41.0% were physically inactive, and 57.3% observed religious fast. The age-standardised prevalence of abdominal obesity was 29.3% in men and 58.5% in women, but that of metabolic syndrome was comparable between men (39.2%) and women (39.0%). The prevalence of diabetes was underestimated if only fasting blood glucose (FBG) was used for the diagnosis compared to combination of FBG and glycated haemoglobin (HbA1c) (6.7% in men and 3.8% in women vs. 12.1% in men and 5.6% in women). More than a quarter (26.1%) of men and 8.7% of women had estimated 10-year risk of cardiovascular disease of 10% or more. This study revealed the high prevalence of NCD metabolic risk factors among the urban public employees in the highland of Ethiopia.

## Introduction

Non-communicable diseases (NCDs) are globally recognized threats to socio-economic developments^1–3^. NCDs are new priorities and additional burdens on health in low- and middle-income countries, where urbanization and lifestyle changes are advancing rapidly^4^. In addition, low birth weight and childhood malnutrition may increase the risks of cardiovascular diseases and diabetes in adulthood^5,6^.

Ethiopia is a low-income country in east Africa: total population is about 102.4 million and 20% of them are urban residents^7^. Although infectious diseases and undernutrition are still prevalent, the burden of NCDs is increasing. Estimated age-standardized death rate of all NCDs was 589.4 per 100,000 population^8^, and NCDs were estimated to account for 30% of total deaths in Ethiopia^9^.

Population-based NCD risk factor surveys sponsored by the World Health Organization (WHO) applying a standardized method, *i*.*e*. STEPwise approach to surveillance (STEPS), that included assessments of behavioural risk factors, anthropometrics and blood pressure, and fasting blood glucose (FBG) and lipids^10^, were conducted three times in the past in Ethiopia^11–14^. However, FBG and lipids were not assessed until the most recent survey in 2015^13,14^. Furthermore, glycated haemoglobin (HbA1c) has never been assessed, although combined use of FBG and HbA1c were recommended to screen individuals with diabetes or prediabetes^15,16^. In addition to the three STEPS surveys, several population-based surveys had been conducted in various regions in the country^17–19^ and in a few workplaces in Addis Ababa^20–22^. The reported prevalence of each NCD risk factor varied: overweight/obesity, less than 3% to more than 25%; hypertension, less than 10% to around 28%; and diabetes, 2.1% to 6.5%.

The previous studies indicated that the prevalence of NCD risk factors was higher in urban dwellers than rural residents, perhaps due to the urbanized lifestyle, such as high-energy diet, physical inactiveness, *etc*. Urban employed workers are likely to lead such lifestyle, because of their possible better-off status. A few studies investigated NCD risk factors of employed workers in Addis Ababa, however, such studies have not been done in major cities in other province.

We conducted a cross-sectional epidemiological study on NCD risk factors applying a modified WHO STEPS procedure in northern Ethiopia. This article aims to describe the prevalence of NCD risk factors among public employees in a regional city in Ethiopia. It also aims to examine whether the prevalence of diabetes defined by the value of HbA1c is consistent with that defined by the level of FBG in this population.

## Results

In total, 1527 employees (869 men and 658 women) of the 18 public offices voluntarily participated in the study. The response rate was about 61%. Pregnant women (n = 22), individuals younger than 25 years old (n = 98), and individuals whose age or gender were unknown (n = 27) were excluded from the data analysis, Finally, the data of 1380 subjects (823 men and 557 women) were statistically analysed.

Table 1 shows demographic and behavioural characteristics. Mean age was 39.5 years. Although the participants were all employed by the public offices, 6.3% of them did not complete primary education and 25.4% earned less than 500 US dollars annually. Only 3.3% of men and 0.5% of women were current smokers. The prevalence of *khat* chewing was 2.6% in men and 0.2% in women. Alcohol drinking was not so prevalent either, as only 4.3% of men and none of women drunk 3 or more standard drinks per day. Very few (0.3%) individuals consumed more than 5 servings of fruits and vegetables per day, and majority (68.7%) had less than 1 serving per day. The prevalence of moderate or high levels of total physical activity (≥600 metabolic equivalent minutes (MET-minutes) per week) was 66.0% in men and 48.7% in women, indicating that one third of men and over half of women led sedentary lifestyle.

**Table 1:** Demographic and behavioural characteristics of participants by gender.

Characteristics	Men	Women	All	*P-value* ^*a*^
Number	823	557	1380	
Age, years				0.040
25–34	33.2%	39.1%	35.6%	
35–44	34.4%	34.8%	34.6%	
45–54	23.8%	18.3%	21.6%	
55–64	8.6%	7.7%	8.3%	
mean (95% CI)^b^	40.3 (39.6–40.9)	38.3 (37.6–39.1)	39.5 (39.0–40.0)	<0.001
Marital status				<0.001
Never married	25.2%	23.5%	24.5%	
Currently married	70.8%	59.6%	66.3%	
Separated/divorced/widowed	4.0%	16.9%	9.2%	
Formal education, years				
≤8	5.7%	7.1%	6.3%	0.249
9–12	12.5%	10.1%	11.5%	
>12	81.9%	82.8%	82.2%	
Annual income, USD per adult^*c*^			<0.001	
<500	21.0%	31.9%	25.4%	
500–1000	28.3%	29.9%	28.9%	
1001–1500	21.7%	17.5%	20.0%	
>1500	29.0%	20.7%	25.7%	
median (IQR)	1142 (1143)	1001 (1001)	990 (1025)	<0.001
Religion				0.459
Ethiopian Orthodox Christian	95.6%	95.1%	95.4%	
Muslim	2.9%	2.5%	2.8%	
Other Christian	1.5%	2.3%	1.8%	
Fasting practice, length of fasting on a fast day			<0.001	
None	52.5%	28.5%	42.8%	
<6 hours	9.3%	5.2%	7.7%	
≥6 hours	38.2%	66.3%	49.6%	
Fruit/vegetable intake, servings per day				<0.001
<1	71.9%	63.9%	68.7%	
1–2.9	23.8%	30.9%	26.6%	
3–4.9	3.9%	5.0%	4.4%	
≥5.0	0.4%	0.2%	0.3%	
Tobacco smoking				<0.001
non-smoker	91.0%	98.9%	94.2%	
ex-smoker	5.7%	0.6%	3.6%	
current smoker	3.3%	0.5%	2.2%	
*Khat* chewing				<0.001
non-chewer	90.7%	99.1%	94.1%	
ex-chewer	6.7%	0.7%	4.3%	
current chewer	2.6%	0.2%	1.6%	
Alcohol drinking, days per week				<0.001
<1 day per month	23.6%	73.9%	42.9%	
1–3 days per month	28.3%	20.3%	25.2%	
1–2 days per week	32.1%	4.7%	21.6%	
≥3 days per week	15.9%	1.1%	10.2%	
Alcohol drinking, standard drinks per day				<0.001
<1	72.2%	97.7%	81.1%	
1–2.9	23.5%	2.3%	16.1%	
3–3.9	3.1%	0.0%	2.0%	
≥4.0	1.2%	0.0%	0.8%	
Physical activity, MET-minutes per week				<0.001
<600	34.0%	51.4%	41.0%	
600–2999	52.1%	37.5%	46.2%	
≥3000	13.9%	11.2%	12.8%	

Abbreviations: CI, confidence interval; USD, United States dollar; IQR, Interquartile range; MET, metabolic equivalent.

^a^Differences between all men and all women were tested with a chi-squared test and t-test as appropriate.

^b^95% CI of the mean.

^c^Annual household income divided by number of household members ≥18 years. (1 USD ≈ 21 Ethiopian Birr).

Table 2 shows the percentages of biological indicators classified by appropriate criteria, and Table 3 shows the prevalence and the age-standardized prevalence of biological NCD risk factors by gender. Overweight/obesity was more prevalent in women (32.8%) than men (28.2%), but underweight was also more common in women (15.3%) than men (10.6%). The prevalence of increased waist circumference and increased waist-hip ratio were 31.1% and 50.9% in men and 59.8% and 48.8% in women, respectively, showing the high prevalence of abdominal obesity. The age-standardized prevalence of hypertension was 22.4% in men and 15.3% in women.

**Table 2:** Physical and biochemical characteristics of participants by gender.

Characteristics	Men	Women	All	*P*-value^a^
Number	823	557	1380	
Body mass index, kg/m^2^				0.001
<18.5	10.6%	15.3%	12.5%	
18.5–24.9	61.2%	51.9%	57.5%	
25.0–29.9	25.1%	27.2%	26.0%	
≥30.0	3.1%	5.6%	4.1%	
mean (95% CI)	23.2 (14.8–31.6)	23.1 (13.9–32.4)	23.2 (14.4–31.9)	0.623
Waist circumference, cm				<0.001
≤80	21.4%	40.3%	29.0%	
81–90	34.0%	26.0%	30.8%	
91–94	13.6%	12.2%	13.0%	
>94	31.1%	21.6%	27.2%	
mean (95% CI)	89.0 (68.6–109.3)	84.3 (59.9–108.7)	87.1 (64.4–109.6)	<0.001
Waist-hip ratio				<0.001
<0.80	7.1%	28.3%	17.4%	
0.80–0.84	15.6%	22.9%	18.5%	
0.85–0.89	26.4%	23.6%	25.3%	
≥0.90	50.9%	25.2%	40.5%	
mean (95% CI)	0.90 (0.75–1.06)	0.85 (0.68–1.02)	0.88 (0.71–1.05)	<0.001
Systolic blood pressure, mmHg				<0.001
<120	44.8%	69.7%	54.9%	
120–129	27.1%	16.0%	22.6%	
130–139	15.9%	6.3%	12.0%	
≥140	12.2%	8.1%	10.5%	
mean (95% CI)	123 (92–154)	114 (82–147)	119 (87–152)	<0.001
Diastolic blood pressure, mmHg				<0.001
<80	44.2%	63.7%	52.1%	
80–84	21.8%	15.4%	19.2%	
85–89	16.6%	10.2%	14.0%	
≥90	17.4%	10.6%	14.7%	
mean (95% CI)	81 (62–101)	77(59–96)	80 (60–99)	<0.001
Fasting blood glucose, mg/dL				<0.001
<100	74.7%	89.2%	80.6%	
100–125	18.6%	7.0%	13.9%	
≥126	6.7%	3.8%	5.5%	
mean (95% CI)^b^	96 (93–98)	88 (86–91)	93 (90–95)	<0.001
HbA1c, %				<0.001
<5.7	55.6%	64.3%	59.1%	
5.7–6.4	32.3%	30.1%	31.4%	
≥6.5	12.1%	5.6%	9.5%	
mean (95% CI)^b^	5.8 (3.5–8.1)	5.6 (3.4–7.8)	5.7 (3.4–8.0)	0.031
Total cholesterol, mg/dL				0.596
<150	25.2%	23.4%	24.5%	
150–189	42.8%	44.8%	43.6%	
190–199	6.5%	7.0%	6.7%	
200–239	18.5%	19.4%	18.9%	
≥240	7.0%	5.4%	6.3%	
mean (95% CI)	176 (99–254)	176 (102–250)	176 (100–252)	0.932
HDL cholesterol, mg/dL				<0.001
<40	70.1%	41.6%	59.2%	
40–49	21.0%	32.5%	25.4%	
≥50	8.8%	25.9%	15.3%	
mean (95% CI)^b^	34 (32–37)	41(39–44)	37 (34–39)	<0.001
LDL cholesterol, mg/dL				<0.001
<100	43.1%	57.7%	48.4%	
100–129	35.2%	25.2%	31.6%	
130–159	15.8%	13.4%	15.0%	
≥160	5.8%	3.6%	5.0%	
mean (95% CI)	107 (44–170)	96 (26–167)	103 (37–170)	<0.001
Triglycerides, mg/dL				<0.001
<100	20.2%	14.3%	17.9%	
100–149	29.3%	21.8%	26.4%	
150–199	18.8%	17.8%	18.4%	
≥200	31.7%	46.1%	37.3%	
mean (95% CI)^b^	154 (151–158)	191 (187–195)	168 (164–171)	<0.001
Haemoglobin, mg/dL				<0.001
<11.0	0.2%	0.4%	0.3%	
11.0–11.9	0.2%	1.8%	0.9%	
12.0–12.9	0.9%	5.4%	2.7%	
13.0–16.9	53.6%	89.6%	68.1%	
≥17.0	45.1%	2.9%	28.0%	
mean (95% CI)	16.7 (14.0–19.5)	14.6 (12.2–17.1)	15.9 (12.5–19.2)	<0.001

Abbreviations: CI, confidence interval; HbA1c, glycated haemoglobin; HDL, high-density lipoprotein; LDL, low-density lipoprotein.

^a^Differences between all men and all women were tested with a chi-squared test or t-test as appropriate.

^b^Log-transformed data were used.

**Table 3:** Prevalence and age-standardised prevalence of non-communicable disease risk factors by gender (%).

Risk factors	Prevalence (95% CI)				Age-standardised prevalence^a^ (95% CI)			
	Men	Women	All	*P-value* ^*b*^	Men	Women	All	*P-value* ^*b*^
Overweight/obesity								
BMI ≥25 kg/m^2^	28.2 (25.2–31.4)	32.8 (29.0–36.8)	30.1 (27.7–32.6)	0.720	26.9 (23.9–29.9)	31.5 (27.9–35.1)	28.5 (26.3–30.8)	0.008
BMI ≥30 kg/m^2^	3.1 (2.0–4.4)	5.6 (3.9–7.7)	4.1 (3.1–5.2)	0.025	2.9 (1.8–4.0)	5.4 (3.6–7.3)	3.9 (2.9–4.9)	0.008
Increased waist circumference Men >94 cm, women >80 cm	31.1 (28.0–34.3)	59.8 (55.8–66.9)	42.7 (40.1–45.3)	<0.001	29.3 (26.3–32.3)	58.5 (54.7–62.3)	40.1 (38.4–43.4)	<0.001
Increased waist-hip ratio Men ≥0.9, women ≥0.85	50.9 (47.4–54.3)	48.8 (44.7–53.0)	50.0 (47.4–52.7)	0.460	47.7 (44.6–50.9)	47.9 (43.9–51.9)	47.7 (45.3–50.2)	0.512
Hypertension SBP/DBP ≥140/90 mmHg or on medication	22.5 (19.7–25.4)	15.3 (12.5–18.4)	19.6 (17.5–21.7)	0.001	22.4 (19.6–25.1)	15.3 (12.4–18.2)	19.4 (17.4–21.4)	0.017
Diabetes								
Fasting blood glucose ≥126 mg/dL or on treatment	7.9 (6.2–9.9)	4.1 (2.7–6.0)	6.4 (5.2–7.8)	0.005	7.2 (5.5–8.9)	4.1 (2.5–5.7)	6.0 (4.8–7.2)	<0.001
HbA1c ≥6.5% or on treatment	12.5 (10.3–14.9)	5.6 (3.9–7.7)	9.7 (8.2–11.3)	<0.001	11.4 (9.4–13.5)	5.6 (3.7–7.4)	9.2 (7.7–10.6)	<0.001
Fasting blood glucose ≥126 mg/dL or HbA1c ≥6.5% or on treatment	13.0 (10.8–15.4)	5.9 (4.2–8.1)	10.1 (8.6–11.8)	<0.001	12.1 (10.0–14.2)	5.9 (4.0–7.8)	9.7 (8.2–11.2)	
Raised total cholesterol ≥190 mg/dL	32.0 (29.0–35.3)	31.8 (28.1–35.8)	31.9 (29.5–34.4)	0.953	30.4 (27.3–33.4)	30.6 (27.0–36.7)	30.3 (28.0–32.6)	0.354
Low HDL cholesterol Men <40 mg/dL, women <50 mg/dL	70.1 (66.9–73.2)	74.1 (70.2–77.9)	71.6 (69.2–74.1)	0.118	69.9 (66.7–73.2)	73.8 (68.9–77.7)	71.3 (68.6–73.8)	0.097
Raised LDL cholesterol ≥130 g/dL	21.6 (18.9–24.7)	17.0 (13.8–21.0)	20.0 (17.8–22.3)	0.520	20.8 (18.0–23.7)	16.0 (12.8–19.2)	18.9 (16.7–21.0)	0.145
Raised triglycerides ≥150 g/dL	50.5 (47.0–54.0)	63.9 (59.7–68.1)	55.7 (53.0–58.3)	<0.001	48.7 (45.3–52.2)	63.2 (59.0–67.4)	54.5 (51.8–57.2)	<0.001
Metabolic syndrome^c^	41.1 (37.7–44.5)	40.2 (36.2–44.3)	40.7 (38.2–43.3)	0.751	39.2 (36.0–42.4)	39.0 (35.1–42.9)	39.0 (36.5–41.5)	0.476
Framingham risk score^d^ intermediate/high risk (≥10%)	22.3 (19.4–25.5)	7.0 (4.9–9.6)	16.5 (14.5–18.7)	<0.001	26.1 (23.8–28.4)	8.7 (6.0–11.4)	19.6 (17.6–21.5)	<0.001

Abbreviations: BMI, body mass index; SBP, systolic blood pressure; DBP, diastolic blood pressure; HbA1c, glycated haemoglobin; HDL, high-density lipoprotein; LDL, low-density lipoprotein.

^a^Age-standardized prevalence was calculated by direct standardization method using the Ethiopian 2007 population and housing census as a standard population.

^b^Differences between all men and all women were tested with a chi-squared test.

^c^Minimum of three of the following criteria: SBP/DBP ≥130/85 mmHg or on medication; fasting blood glucose ≥100 mg/dL or on treatment; triglycerides ≥150 mg/dL or on medication; HDL cholesterol <40 mg/dL for men and <50 mg/dL for women; waist circumference ≥94 cm for men and ≥80 cm for women.

^d^10-year cardiovascular disease risk prediction for the age 30 years old and more based on the Framingham cardiovascular disease risk prediction point score method.

The mean value of total cholesterol was 176 mg/dL both in men and women, and the mean value of high-density lipoprotein (HDL) cholesterol was as low as 34 mg/dL in men and 41 mg/dL in women, while the mean value of triglycerides was 154 mg/dL in men and 191 mg/dL in women, reaching the borderline-high level. The age-standardized prevalence of raised total cholesterol was 30.4% in men and 30.6% in women, that of low HDL cholesterol was 69.9% in men and 73.8% in women, and that of raised low-density lipoprotein (LDL) cholesterol was 20.8% in men and 16.0% in women. The prevalence of high triglycerides (≥200 mg/dL) was 31.7% in men and 46.1% in women.

The age-standardized prevalence of diabetes was 12.1% in men and 5.9% in women. The prevalence of diabetes defined only by the level of FBG (≥126 mg/dL) was 6.7% in men and 3.8% in women.

**Figure 1:**
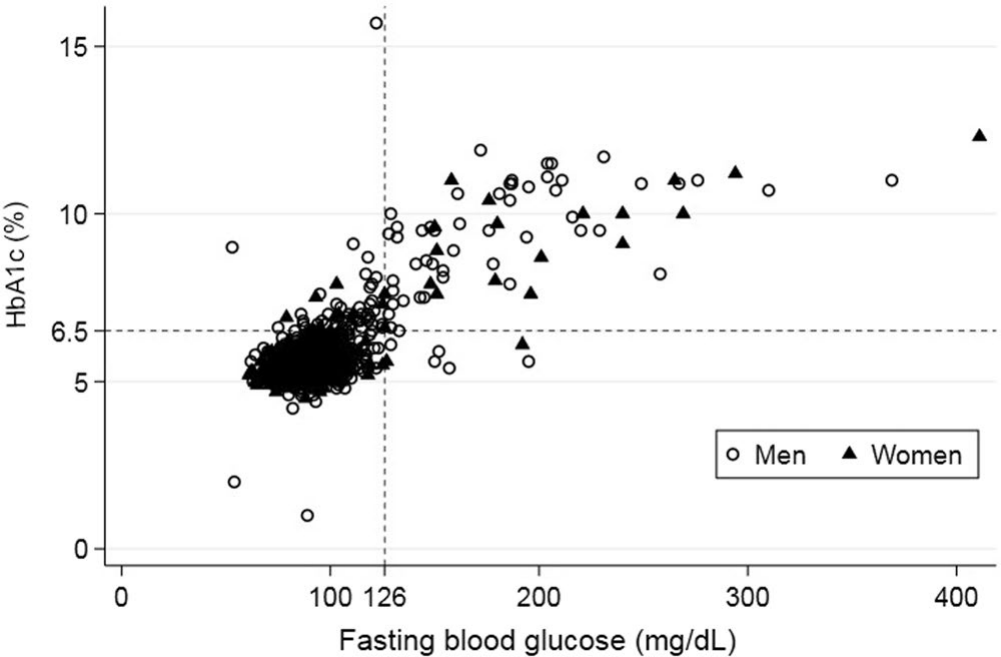
Scatter plot of fasting blood glucose (FBG) and glycated haemoglobin (HbA1c) values. Four quadrants divided by cut-off lines, FBG ≥126 mg/dL and HbA1c ≥6.5%, for detecting diabetes display the interrater agreement and disagreement of the detection by these two methods.

Figure 1 displays a scatter plot of FBG and HbA1c values with cut-off lines (FBG ≥126 mg/dL, HbA1c ≥6.5%) for the detection of diabetes. The interrater agreement of the detection by these two methods was 93.3% (kappa = 0.632, *p* < 0.001) in men and 97.1% (kappa = 0.689, *p* < 0.001) in women. Correlation coefficients between FBG and HbA1c values were 0.767 for men and 0.829 for women, respectively.

The age-standardized prevalence of metabolic syndrome was as high as 39.0%, implying that this population was prone to develop cardiovascular diseases. The Framingham risk score indicated that 26.1% of men and 8.7% women are with the intermediate/high risk of developing cardiovascular diseases in 10-years.

Table 4 shows the prevalence of NCD risk factors among religious fast-observers and non-fast-observers. The prevalence of diabetes, raised LDL cholesterol, and raised triglycerides were significantly lower in fast-observers than non-fast-observers in men, whereas such significant difference was not shown in women. The prevalence of overweight/obesity, increased waist circumference, increased waist-hip ratio, low HDL cholesterol, and raised triglycerides were not significantly different in both men and women.

**Table 4:** Prevalence of non-communicable disease risk factors among religious fast-observers and non-fast-observers (%).

	Men			Women			All		
	Fast-observer	Non-fast-observer	*P- value*	Fast-observer	Non-fast-observer	*P- value*	Fast-observer	Non-fast-observer	*P- value*
N	377	438		394	161		771	599	
Body mass index, kg/m^2^			0.047			0.313			0.081
<18.5	13.3	8.0		15.0	16.3		14.2	10.3	
18.5–24.9	61.6	60.9		50.4	55.6		55.9	59.5	
25.0–29.9	22.4	27.6		28.0	25.0		25.3	26.9	
≥30.0	2.7	3.4		6.6	3.1		4.7	3.4	
Increased waist circumference Men >94 cm, women >80 cm.	31.8	36.2	0.186	62.8	58.5	0.344	47.6	42.2	0.045
Increased waist-hip ratio Men ≥0.9, women ≥0.85	48.0	53.6	0.140	50.0	45.9	0.385	49.0	51.5	0.361
Hypertension^a^	21.8	23.3	0.601	15.7	14.3	0.667	18.7	20.9	0.311
Diabetes^b^	9.3	16.2	0.003	6.3	5.0	0.534	7.8	13.2	0.001
Fasting blood glucose ≥126 mg/dL	4.5	8.8	0.006	3.8	3.7	0.285	4.2	7.4	0.001
HbA1c ≥6.5%	8.5	15.2	0.001	5.8	5.0	0.658	7.1	12.5	0.001
Low HDL cholesterol Men <40 mg/dL, women <50 mg/dL	67.3	72.2	0.130	72.4	78.9	0.135	69.8	73.9	0.103
Raised LDL cholesterol ≥130 mg/dL	18.6	24.4	0.055	16.9	17.7	0.829	17.8	22.8	0.030
Raised triglycerides ≥150 mg/dL	44.1	55.7	0.001	64.3	63.2	0.808	50.4	57.6	0.198

Abbreviations: HDL, high-density lipoprotein; LDL, low-density lipoprotein; HbA1c, glycated haemoglobin.

^a^Systolic blood pressure/diastolic blood pressure ≥140/90 mmHg or on medication.

^b^Fasting blood glucose ≥ 126 mg/dL or HbA1c ≥6.5% or on treatment.

## Discussion

This study comprehensively surveyed the prevalence of various NCD risk factors among public employees in a regional city in northern Ethiopia. This is the first epidemiological study that measured HbA1c targeting apparently healthy population in Ethiopia. Our findings showed that the prevalence of diabetes defined by both FBG and HbA1c values was much higher than the WHO estimated diabetes prevalence (men 4.0%; women 3.6%)^23^ and the prevalence of the impaired fasting glucose and diabetes (≥110 mg/dL) of the 2015 STEPS (men 5.1%; women 5.7%)^13^, particularly in men. However, strict comparison would not be possible as the age-range of the survey participants and sampling methods differed; *i*.*e*., 15–69 years of age and probability random sampling in the STEPS and 25–64 years of age and worksite volunteers in the present study. The prevalence of diabetes defined only by the level of FBG was comparable with the findings of the previous population-based studies^17,20^.

Discrepancy between the values of HbA1c and FBG sometimes happens due to various reasons, such as anaemia and ethnicity^24–27^. However, HbA1c values of this population were unlikely to be affected by anaemia, as the prevalence of anaemia was only 0.3%. Furthermore, we used WHO-recommended HbA1c cut-off^28^, which is reported to be reliable for any population groups. The sensitivity/specificity and positive/negative predictive values of FBG and HbA1c for diagnosing diabetes cannot be shown because of lack of the standard diagnostic values measured by the oral glucose tolerance test. Further studies of this population on blood glucose, HbA1c, and glucose tolerance is required.

Although appropriate HbA1c cut-off value of this population has not been determined yet, substantial portion of individuals with diabetes might have been undetected, if only FBG was used for screening. In our study, criteria using only FBG missed 61 subjects (4.4%), whereas HbA1c missed only 7 subjects (0.5%) (Supplementary Table 1). While HbA1c values are stable reflecting the blood glucose levels in the past several weeks, FBG values fluctuate depending on the condition of the individual at the time of blood sampling. Since the majority of the study participants observed annual and weekly fast, blood samples might be taken after a very long fasting.

The FBG readings might be decreased because of the use of a portable blood glucose analyser and whole blood samples^24,29,30^. The performance of the portable analysers was reported to be inconsistent in the presence of low oxygen tension and an increased number of red blood cells, both common phenomena in the highlands. The FBG reading of the whole blood measured by the portable analysers may shift lower than that of the plasma measured by the laboratory equipment, when the haematocrit level of the sample is very high. Another limitation of the portable analysers is that they can measure values within a certain range, thus values higher or lower than the range are regarded as missing data. However, portable analysers have been commonly used by the WHO NCD risk factor surveys^10^, and was used for the 2015 STEPS survey in Ethiopia^14^.

This study showed that the prevalence of low HDL cholesterol was as high as about 71.3%, consistent with the findings of the 2015 STEPS survey^13,14^. The prevalence of elevated triglycerides was about 55%, twice as much higher than that of the 2015 STEPS survey. The observation indicates high prevalence of atherogenic dyslipidaemia in the urban workers characterized by low HDL cholesterol, high triglycerides, and implied existence of high small-dense LDL cholesterol levels. Prevalent abdominal obesity would explain the present finding, but several other factors, such as physical inactivity and very high carbohydrate intake (>60% of total energy intake)^31^, might have contributed to the low HDL cholesterol and elevated triglycerides. Elevated triglycerides among highlanders living about 4000 m above sea level were reported, since triglycerides would be a fast energy source for living in a harsh environment^32,33^. However, the altitude of the study area was not so high that elevation of triglycerides was unlikely to occur. Very high carbohydrate intake might be a contributing factor, considering that the majority of the study participants had very little amount of vegetables and fruits and frequently abstained animal products. The prevalence of raised triglycerides was higher in women than men, partly because women might eat less animal products than men. Further studies on foods and nutrition are needed to explain the causes of the high prevalence of dyslipidaemia.

Our findings indicated that the prevalence of overweight/obesity was much higher than that of the 2015 STEPS survey (men 4.4%; women 8.8%; urban areas 12.7%)^14^ and WHO estimation (men 11.4%; women 28.3%)^23^, and abdominal obesity was highly prevalent. Although one possibility for the explanation would be difference in the target population (probability random sampling of the general population vs. civil servants in a regional city), the present finding at least indicates threat of obesity is rapidly increasing in middle-aged working population of Ethiopia. The prevalence of underweight was still at a substantial level in both men and women, although it was less than that of the 2015 STEPS survey (men 23.3%; women 19.4%). This indicates a dual burden of NCDs and undernutrition, therefore, both overweight/obesity and underweight should be paid attention simultaneously, particularly for women.

The age-standardized prevalence of hypertension in men and women was similar to the findings of the 2015 STEPS survey (men 15.7%; women 16.5%)^13^, and a community-based study in Mekelle (men 22.5%; women 19.0%)^18^. The hypertension prevalence was higher than that in rural areas^11,14^, but lower than that in Addis Ababa^12,22^.

We found that the prevalence of diabetes, raised LDL cholesterol, and raised triglycerides were significantly lower in fast-observers than non-fast-observers in men, whereas such significant difference was not seen in women. Decreased levels of blood glucose and improved lipid profile were previously reported among fast-observers of Eastern Orthodox and Coptic Christians^34–36^, who observe fasting period similar to Ethiopians but are allowed to have seafood. This is the first report suggesting the possible health impact of the strict vegetarian-style fast observed by Ethiopian Orthodox Christians. However, since the period of blood sampling included a long-term annual fast period, weekly-fast days, and non-fast periods/days, this might not have related to the fast directly, but to other confounding factors such as well-regulated lifestyle of fast-observers. Further studies are required to identify health impacts of the fast observed by Ethiopian Orthodox Christians.

The prevalence of *khat* chewing in men and women was much less than the findings of the 2015 STEPS survey (21.1% in men and 9.4% in women)^14^. In Ethiopia, *khat* chewing is commonly practiced among Muslims^37^. The low prevalence might be due to the predominantly Ethiopian Orthodox Christian population, as well as the low *khat* production in the study area.

The prevalence of metabolic syndrome was as high as around 40%. Since the target population was relatively young, the Framingham risk score for 10-year risk of cardiovascular diseases was not so high comparing to the high prevalence of metabolic syndrome. The cardiovascular disease risk of this population is likely to increase with age, unless they improve their lifestyle^38^.

Although this study examined only public employees in a regional city, we may draw public health policy implications from the study findings. First, the burden of NCDs may be higher than expected, as increased levels of blood glucose and dyslipidaemia might have been under detected. NCD risk factors, including HbA1c, HDL and LDL cholesterol and triglycerides, need to be surveyed regularly. Most of the study participants had never been examined the risk factors before, therefore, regular health check-up or screening mechanisms need to be introduced. Second, future risks of cardiovascular diseases are likely to increase, unless proper interventions started immediately. Metabolic syndrome, abdominal obesity, low HDL cholesterol and elevated triglycerides were highly prevalent, indicating increased risk of cardiovascular diseases. Practical strategies including health education interventions to modify lifestyle is urgently required. Third, NCD prevention need to be integrated into the current primary health care activities. As shown in the substantial levels of the underweight prevalence, a dual burden of NCDs and undernutrition exists. In addition, our previous qualitative study found that taking lots of salt was perceived as good for health because of iodine deficiency prevention campaign, neglecting the risk of hypertension^39^. Therefore, it is needed to develop a comprehensive strategy to prevent both NCDs and undernutrition.

The strength of this study was that it was the first epidemiological study assessed HbA1c targeting apparently healthy population in Ethiopia, and showed that the prevalence of diabetes was higher than expected. This study also contributed to identify an increased risk of cardiovascular diseases among urban public employees in northern Ethiopia. We showed that low HDL cholesterol, elevated triglycerides, and abdominal obesity were highly prevalent. The increased risk of cardiovascular diseases was indicated by the high prevalence of metabolic syndrome, as well as Framingham risk score, calculated for the first time in epidemiological studies targeting apparently healthy population in Ethiopia.

However, this study has several limitations. First, we targeted only public employees in a regional capital city, which may not represent the nationwide urban situation. Second, the study subjects were sampled based on voluntary participation, therefore, health conscious individuals might be over-represented. Caution is required when we compared the findings with those of the 2015 STEPS, which applied a probability sampling procedure. However, a relatively large sample size of the study was likely to make the findings reliable. Third, we used portable blood analysers, thus could not measure values lie outside the measuring ranges of each analyser. While these analysers were properly calibrated, measuring whole blood instead of blood plasma might have affected the readings, as our targets were highlanders. However, previous STEPS surveys used portable analysers and measured whole blood as the WHO standard protocol. Fourth, due to the cross-sectional nature of this study, causal associations cannot be inferred.

In conclusion, the current survey revealed a high prevalence of NCD risk factors among the urban public employees in northern Ethiopia. Metabolic syndrome, abdominal obesity, low HDL cholesterol and elevated triglycerides were highly prevalent, indicating increased risk of cardiovascular diseases. NCD risk factors of most of the study participants had never been examined before, therefore, a regular health check-up mechanism needs to be introduced. Health education interventions to modify the urbanized lifestyle is also required.

## Methods

### Study site

We conducted this study in Mekelle, the capital city of Tigray province in northern Ethiopia, located about 780 km north of Addis Ababa. The altitude is around 2000 m above sea level. Estimated population in 2016 was about 320,000^40^, and the public administration employed about 5% of the total population^41^. We chose Mekelle, as it is one of the most populous cities in Ethiopia and a major city in the northern region, but any epidemiological studies of NCD risk factors among urban employed workers have not conducted before.

### Target population and sampling

We defined the target population of this study as adults between 25 and 64 years of age, excluding pregnant women, who were employed by the public administration offices in Mekelle. Total number of the public employees in the city was estimated to be about 16,000^40,41^. From previous studies^19,20,22^, we assumed the prevalence of hypertension to be 28% and that of diabetes to be 8%. The necessary sample sizes to obtain single population proportions with the 95% confidence interval widths of 5% for hypertension and 3% for diabetes were 1,240 for hypertension and 1,257 for diabetes. Since the present study also aimed to describe prevalence of other variables, we set the target sample size to be 1,500 (approximately 10% of the entire worksite population).

We purposively selected 12 provincial offices and six city offices of various types of public services, which agreed to let their employees voluntarily participate in the study. Although we could not apply a random sampling strategy due to lack of access to reliable records of employment registration, we aimed to choose a wide range of workers in public services, including offices of regional council, judicial administration, finance, infrastructure, labour, agriculture, industrial development, health, and social services. The estimated total number of the employees in the 18 offices was about 2500.

### Data collection

The study was conducted from October 2015 to February 2016. We used a modified WHO STEPS questionnaire^10^, to which we added questions about *khat* chewing and religious fast-observing. We conducted a qualitative study prior to this epidemiological study, and incorporated its preliminary findings to the questionnaire^39^. *Khat* is a plant containing amphetamine-like stimulant. Many local people observe long-term annual fast-periods and weekly fast-days, in total around a half year, in which they abstain any kind of animal products for a whole day and any foods and drinks from midnight to three o’clock in the afternoon. The questionnaire developed in English was translated into local language, Tigrigna, pretested and revised, incorporating data collectors’ feedback.

Three male and five female nurses with college degree and at least five-year clinical experience were recruited as data collectors. They were trained for five days on interview skills, the standard physical measurements following the WHO guideline, and blood test procedures using portable analysers. During the training, the eight data collectors conducted interviews, physical measurements, and blood tests to each two volunteers. The survey procedure was modified according to the feedback of data collectors and volunteers during the training. Two supervisors monitored the quality of the data collection.

We set up a temporary study clinic in a conference room in each office. First, about 40 μl of capillary blood was sampled from a fingertip after at least eight-hour fasting, and analysed using portable analysers (Accu-Check Performa, Roche Diagnostics, Indianapolis, IN, USA, for glucose; cobas b 101, Roche Diagnostics, Indianapolis, IN, USA, for total and HDL cholesterol, triglycerides and HbA1c; Hb 201+, HemoCu, Ängelholm, Sweden, for haemoglobin) Then, physical measurements and questionnaire-based interviews were conducted. Height, weight, waist and hip circumferences, were measured in light clothing without shoes. Blood pressure was measured three times in the right upper arm by an automatic digital sphygmomanometer (HEM-7200, OMRON, Kyoto, Japan). Systolic blood pressure (SBP), diastolic blood pressure (DBP), and pulse per minute were recorded, and the arithmetic mean of the second and third readings of blood pressure was used for the analysis.

### NCD risk factors

The below listed cut-off values of NCD risk factors were used for the statistical analysis.

- Body mass index (BMI): weight in kilograms divided by height in meters squared^42^ underweight: BMI <18.5 kg/m^2^; overweight: BMI 25–29.9 kg/m^2^; obesity: BMI ≥30 kg/m^2^
- Increased waist circumference: >94 cm for men; >80 cm for women^43^
- Increased waist-hip ratio: ≥0.90 for men; ≥0.85 for women^43^
- Hypertension: SBP ≥140 mmHg or DBP ≥90 mmHg or on antihypertensive medication^44^
- Diabetes: FBG ≥126 mg/dL or HbA1c ≥6.5% or on diabetes treatment^28,45^
- Raised total cholesterol: ≥190 mg/dL^46^
- Low HDL cholesterol: <40 mg/dL for men; <50 mg/dL for women^31^
- Raised LDL cholesterol: ≥130 mg/dL^31^
- Borderline-high triglycerides: ≥150 mg/dL; high triglycerides: ≥200 mg/dL^31^
- Anaemia: haemoglobin <11 mg/dL^47^
- Physical inactivity: <600 MET-minutes per week^48^
- Low fruit and vegetable consumption: <5 servings per day^49^
- Alcohol: ≥4 standard drinks per day for men; ≥3 standard drinks per day for women^50^
- Metabolic syndrome: at least three items of the followings^51^

- Waist circumference ≥94 cm for men; ≥80 cm for women
- SBP ≥130 mmHg or DBP ≥85 mmHg
- FBG ≥100 mg/dL
- Triglycerides ≥150 mg/dL
- HDL cholesterol <40 mg/dL for men; <50 mg/dL for women

- The Framingham risk score for estimating the 10-year cardiovascular disease risk of an individual: calculated from the sum of gender-specific scores of age, smoking, SBP, diabetes, total cholesterol, and HDL cholesterol^52^ intermediate/high risk: ≥10%.

### Data analysis

All continuous readings of physical and biochemical measurements were categorized into several groups according to common standards^31,42–50^. All of the statistical analyses were performed using SPSS Version 23.0. Log-transformed values of FBG, HbA1c, HDL cholesterol, and triglycerides were used for calculating means and 95% confidence intervals. Chi-squared test was applied to test the difference between men and women on each categorical data, and Student’s t-test and ANOVA were used for testing differences of means. Age-standardized prevalence rates were calculated by the direct standardization method using the Ethiopian 2007 population and housing census as a standard population^40^. Cohen’s kappa coefficients were calculated for the detection of diabetes by FBG and HbA1c; correlation coefficients between values of FBG and HbA1c were also calculated.

### Ethical considerations

This study was approved by the Bioethics Review Committee of Nagoya University School of Medicine, Japan (approval no. 2014–0107), and the Institutional Review Board of Mekelle University College of Health Science, Ethiopia. Written informed consent was obtained from all participants after adequate explanations of the study. The study was conducted according to the Declaration of Helsinki, and the Ethical Guidelines for Medical and Health Research Involving Human Subjects, enforced by the Ministry of Health, Labour and Welfare, Government of Japan.

## Electronic supplementary material

Supplementary Table.
