# Supplementary material for: Non-communicable disease risk factor profile among public employees in a regional city in northern Ethiopia
Source: Sci Rep. 2018 Jun 18;8:9298. doi: 10.1038/s41598-018-27519-6 (PMC6006379; doi:10.1038/s41598-018-27519-6)
Supplement: Supplementary file 1 — Supplementary Table. [file 41598_2018_27519_MOESM1_ESM.docx]

**Non-communicable disease risk factor profile among public employees in a regional city in northern Ethiopia**

Lemlem Weldegerima Gebremariam ^1^

Chifa Chiang ^1^

Hiroshi Yatsuya ^1, 2^

Esayas Haregot Hilawe ^1, 3^

Alemayehu Bayray Kahsay ^4^

Hagos Godefey ^5^

Loko Abraham ^4, 6^

Yoshihisa Hirakawa ^1^

Hiroyasu Iso ^7^

Atsuko Aoyama ^1*^

^1^ Department of Public Health and Health Systems, Nagoya University School of Medicine, Nagoya, Japan

^2^ Department of Public Health, Fujita Health University School of Medicine, Toyoake, Aichi,

Japan

^3^ Tigray Health Research Institute, Mekelle, Ethiopia

^4^ College of Health Sciences, Mekelle University, Mekelle, Ethiopia

^5^ Tigray Regional Health Bureau, Mekelle, Ethiopia

^6^ Ethiopian Pharmaceuticals Fund and Supply Agency, Addis Ababa, Ethiopia

^7^ Public Health Graduate School of Medicine, Osaka University, Suita, Osaka, Japan

**Supplementary Table.**

**Cross-tabulation of fasting blood glucose and glycated haemoglobin (HbA1c)**

|  |  | sample numbers  (%) | | HbA1c | |
| --- | --- | --- | --- | --- | --- |
|  |  |  |  | ≤ 6.4 % | ≥ 6.5 % |
|  | total | | 1372  (100 %) | 1242  (90.5%) | 130  (9.5%) |
| Fasting  blood  glucose | ≤ 125 mg/dL | 1296  (94.5 %) | | 1235  (90.0%) | 61  (4.4%) |
|  | ≥ 126 mg/dL | 76  (5.5 %) | | 7  (0.5%) | 69  (5.0%) |
